# Trends in Postpartum Depressive Symptoms — 27 States, 2004, 2008, and 2012

**DOI:** 10.15585/mmwr.mm6606a1

**Published:** 2017-02-17

**Authors:** Jean Y. Ko, Karilynn M. Rockhill, Van T. Tong, Brian Morrow, Sherry L. Farr

**Affiliations:** ^1^Division of Reproductive Health, National Center for Chronic Disease Prevention and Health Promotion, CDC; ^2^Oak Ridge Institute for Science and Education, U.S. Department of Energy; ^3^Division of Birth Defects and Developmental Disabilities, National Center on Birth Defects and Developmental Disabilities, CDC.

Postpartum depression is common and associated with adverse infant and maternal outcomes (e.g., lower breastfeeding initiation and duration and poor maternal and infant bonding) (*1*–*3*). A developmental *Healthy People 2020* objective is to decrease the proportion of women delivering a live birth who experience postpartum depressive symptoms (PDS).* To provide a baseline for this objective, CDC sought to describe self-reported PDS overall, by reporting state, and by selected sociodemographic factors, using 2004, 2008, and 2012 data from the Pregnancy Risk Assessment Monitoring System (PRAMS). A decline in the prevalence of PDS was observed from 2004 (14.8%) to 2012 (9.8%) among 13 states with data for all three periods (p<0.01). Statistically significant (p<0.05) declines in PDS prevalence were observed for eight states, and no significant changes were observed for five states. In 2012, the overall PDS prevalence was 11.5% for 27 states and ranged from 8.0% (Georgia) to 20.1% (Arkansas). By selected characteristics, PDS prevalence was highest among new mothers who 1) were aged ≤19 years or 20–24 years, 2) were of American Indian/Alaska Native or Asian/Pacific Islander race/ethnicity, 3) had ≤12 years of education, 4) were unmarried, 5) were postpartum smokers, 6) had three or more stressful life events in the year before birth, 7) gave birth to term, low-birthweight infants, and 8) had infants requiring neonatal intensive care unit admission at birth. Although the study did not investigate reasons for the decline, better recognition of risk factors for depression and improved screening and treatment before and during pregnancy, including increased use of antidepressants, might have contributed to the decline. However, more efforts are needed to reduce PDS prevalence in certain states and subpopulations of women. Ongoing surveillance and activities to promote appropriate screening, referral, and treatment are needed to reduce PDS among U.S. women.

PRAMS is an ongoing, population-based surveillance system that collects state-specific data on maternal attitudes and experiences before, during, and soon after pregnancy among women who had a live birth during the preceding 2–9 months.^†^ From year to year, PRAMS survey results are reported by varying numbers of states, New York City, and those areas of New York state outside of New York City (all of which, for simplicity, are referred to as “states” in this report).

For each reporting state, a monthly stratified PRAMS sample of 100–300 new mothers was selected systematically from birth certificates. States that met response rate thresholds for the three periods (≥70% for 2004, ≥65% for 2008, and ≥60% for 2012) were included in this analysis; the thresholds reflect PRAMS data quality goals and changing operational and general national survey response environments over time. The 2012 PRAMS sample represented 1,610,767 women from 27 reporting states and 41% of U.S. births.

Self-reported PDS was ascertained through five responses (“always,” “often,” “sometimes,” “rarely,” and “never”) to the following two questions: 1) “Since your new baby was born, how often have you felt down, depressed, or hopeless?” and 2) “Since your new baby was born, how often have you had little interest or little pleasure in doing things?” Women responding “always” or “often” to either question were classified as experiencing PDS. In 2004 and 2008, these two questions were optional and included in 17 and 22 state surveys, respectively; in 2012, these questions were required for all 27 participating PRAMS states.

Annual PDS prevalence estimates and 95% confidence intervals were calculated for all states with available data, for the 13 states with data for all three periods (Alaska, Colorado, Georgia, Hawaii, Maine, Maryland, Minnesota, Nebraska, Oregon, Rhode Island, Utah, Vermont, and Washington) and for each individual reporting state. Combined and state-specific linear trends over time were assessed using logistic regression models that included birth year and state variables to account for baseline state-specific differences in prevalence. To estimate the average annual change in the prevalence of PDS during 2004–2012, the percentage-point change was calculated using the beta coefficient of the infant’s birth year from the models. Associations between PDS and maternal characteristics (maternal age, race/ethnicity, education, marital status, number of previous live births, and postpartum smoking status), experiences (number of stressful life events experienced in the 12 months before birth), and infant outcomes (gestational age and birthweight and infant neonatal intensive care unit [NICU] admission) were assessed with chi-square tests using 2012 data. In addition, annual percentage-point changes in the prevalence of PDS during 2004–2012 were calculated by selected characteristics. Analyses were conducted using statistical software to account for the complex survey design. Differences with p-values of <0.05 were considered significant.

On average, the PRAMS surveys were completed 125 days after delivery (range = 60–270 days); timing of survey completion did not differ by PDS status. Among states with available data, the prevalence of self-reported PDS declined from 15.5% in 2004 to 13.6% in 2008 and to 11.5% in 2012 (linear trend p<0.01) (Figure) (Table 1). The overall decline was consistent with the changes among the 13 states with data for all three periods; PDS prevalence declined from 14.8% in 2004 to 12.6% in 2008 to 9.8% in 2012 (linear trend p<0.01). The estimated annual percentage-point change during 2004–2012 was -0.6% for all states and for the 13 states with data for all three periods (Table 1). Statistically significant declines in prevalence were observed in eight of 13 states (Alaska, Colorado, Georgia, Hawaii, Minnesota, Nebraska, Utah, and Washington). No statistically significant changes in prevalence were observed in five states (Maine, Maryland, Oregon, Rhode Island, and Vermont); for three states (Maryland, Oregon, and Vermont), prevalence estimates decreased at each period, but did not reach statistical significance.

**FIGURE:**
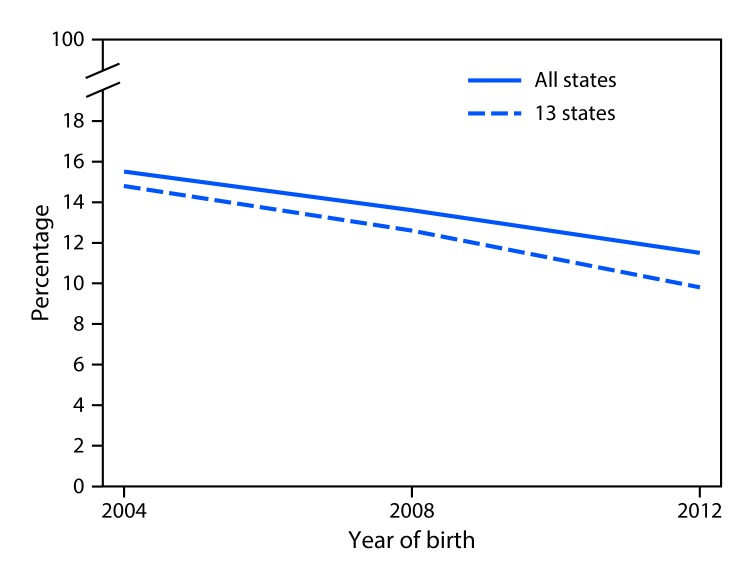
Percentage of new mothers with postpartum depressive symptoms — Pregnancy Risk Assessment Monitoring System (PRAMS) reporting states,* 2004, 2008, 2012^†,§^ * From year to year, PRAMS survey results are reported by varying numbers of states, New York City, and those areas of New York state outside of New York City (all of which, for simplicity, are referred to as “states” in this report). ^†^ The overall trend includes states with data for any period. Thirteen states had data for all three periods: Alaska, Colorado, Georgia, Hawaii, Maine, Maryland, Minnesota, Nebraska, Oregon, Rhode Island, Utah, Vermont, and Washington. ^§^ Significant linear trend assessed using logistic regression model, which included birth year and state variables to account for baseline state-specific differences in prevalence.

**TABLE 1:** Percentage of new mothers with postpartum depressive symptoms, by reporting state — Pregnancy Risk Assessment Monitoring System (PRAMS), United States, 2004, 2008, and 2012

Reporting states	2004 (17 states)	2008 (22 states)	2012 (27 states)	Linear trend* p-value	Average annual percentage-point change from 2004 to 2012^†^
	% (95% CI)	% (95% CI)	% (95% CI)		
**All 27 states**	**15.5 (14.8–16.3)**	**13.6 (12.9–14.3)**	**11.5 (11.0–12.0)**	**<0.01**	**-0.6**
**13 states^§^**	**14.8 (13.9–15.6)**	**12.6 (11.7–13.5)**	**9.8 (9.1–10.6)**	**<0.01**	**-0.6**
Alaska	16.6 (14.2–19.3)	13.1 (10.9–15.6)	12.2 (9.9–14.9)	0.02	-0.5
Arkansas	—^¶^	—^¶^	20.1 (16.1–24.9)	—**	—^††^
Colorado	15.0 (12.8–17.4)	13.4 (11.5–15.5)	8.9 (7.0–11.3)	<0.01	-0.7
Delaware	—^§§^	14.3 (12.4–16.4)	13.6 (11.6–15.9)	—**	—^††^
Georgia	17.2 (14.8–20.0)	12.7 (9.8–16.3)	8.0 (6.1–10.3)	<0.01	-1.1
Hawaii	16.8 (15.3–18.5)	14.5 (12.9–16.3)	10.6 (8.8–12.7)	<0.01	-0.8
Illinois	—^¶^	—^¶^	8.1 (6.5–10.1)	—**	—^††^
Maine	11.1 (9.2–13.4)	12.6 (10.5–15.1)	10.5 (8.1–13.6)	0.76	—^††^
Maryland	15.2 (12.7–18.2)	13.4 (11.1–16.2)	12.1 (9.8–14.9)	0.11	—^††^
Massachusetts	—^§§^	12.7 (10.8–15.0)	11.9 (10.0–14.2)	—**	—^††^
Minnesota	12.7 (10.7–15.0)	9.8 (8.2–11.6)	9.3 (7.4–11.5)	0.03	-0.4
Missouri	—^§§^	—^§§^	14.9 (12.3–17.8)	—**	—^††^
Nebraska	14.3 (12.5–16.2)	10.8 (9.1–12.7)	11.1 (9.1–13.4)	0.03	-0.4
New Jersey	—^¶^	—^¶^	9.7 (8.0–11.7)	—**	—^††^
New Mexico	19.5 (17.4–21.7)	—^§§^	14.0 (11.8–16.6)	—**	—^††^
New York^¶¶^	14.5 (12.0–17.5)	12.6 (10.3–15.2)	—^§§^	—**	—^††^
New York City	—^¶^	—^§§^	11.8 (9.9–14.0)	—**	—^††^
North Carolina	17.7 (15.4–20.2)	14.0 (12.1–16.2)	—^§§^	—**	—^††^
Ohio	—^§§^	16.3 (13.9–19.0)	13.2 (11.2–15.3)	—**	—^††^
Oklahoma	—^¶^	—^¶^	14.9 (12.3–18.0)	—**	—^††^
Oregon	13.2 (11.0–15.74)	12.3 (10.0–14.9)	9.5 (6.9–12.8)	0.06	—^††^
Pennsylvania	—^§§^	11.9 (9.9–14.2)	12.3 (9.9–15.1)	—**	—^††^
Rhode Island	13.4 (11.5–15.6)	13.6 (11.5–16.0)	13.9 (11.9–16.1)	0.75	—^††^
South Carolina	19.6 (16.4–23.2)	—^§§^	—^§§^	—**	—^††^
Tennessee	—^§§^	21.1 (17.5–25.2)	17.0 (14.1–20.5)	—**	—^††^
Utah	14.8 (13.1–16.6)	12.4 (10.8–14.2)	11.3 (9.1–13.3)	0.01	-0.4
Vermont	12.2 (10.3–14.4)	11.6 (9.8–13.8)	10.1 (8.4–12.1)	0.13	—^††^
Washington	13.5 (11.4–16.0)	13.4 (11.3–15.9)	10.1 (7.9–12.5)	0.03	-0.4
Wisconsin	—^§§^	13.5 (11.4–16.1)	11.1 (8.9–13.8)	—**	—^††^
Wyoming	—^§§^	11.6 (9.4–14.3)	13.8 (10.7–17.6)	—**	—^††^

**Abbreviation:** CI = confidence interval.

* State-specific linear trends were assessed using logistic regression models among states with all three periods using year of birth as the predictor. Overall linear trends for all states and for combined 13 states with data for all three periods also were adjusted for state in regression models.

^†^ Average annual percentage-point change during 2004–2012 was calculated using the beta coefficient of the infant’s birth year from the linear model and the average percentage over 2004–2012.

^§^ Included 13 states that had data for all three periods: Alaska, Colorado, Georgia, Hawaii, Maine, Maryland, Minnesota, Nebraska, Oregon, Rhode Island, Utah, Vermont, and Washington.

^¶^ PRAMS state did not ask the postpartum depressive symptoms questions on the survey that year.

** Insufficient data (<3 years) to assess linear trend.

^††^ Annual percentage point-change was not computed because of either nonsignificant linear trend or insufficient data to calculate linear trend.

^§§^ States did not participate in PRAMS or participated in PRAMS but did not meet response rate threshold for that year for data to be included.

^¶¶^ Areas of New York state outside of New York City.

In 2012, the overall prevalence of PDS was 11.5%, representing 184,828 women with PDS in the 27 reporting states. In 2012, state-specific PDS ranged from 8.0% in Georgia to 20.1% in Arkansas (Table 1). In 2012, by selected characteristics, PDS prevalence was highest among the following: women who 1) were aged ≤19 years and 20–24 years (age group), 2) were American Indian/Alaska Natives or Asian/Pacific Islanders (race/ethnicity), 3) had ≤12 years of education (education level), 4) were unmarried (marital status), 5) were postpartum smokers (smoking status), 6) had three or more stressful life events in the year before birth (number of stressful life events), 7) gave birth to term, low-birthweight infants (gestational age and weight), and 8) had infants requiring NICU admission at birth (NICU status) (p<0.05 for all) (Table 2). Notably from 2004 to 2012, PDS prevalence did not significantly decline among American Indian/Alaska Native women and women with term, low-birthweight infants (p>0.05), with PDS prevalence remaining above 17% in 2012.

## Discussion

In this population-based sample of postpartum women, a decline in the prevalence of self-reported PDS was observed from 2004 to 2012 overall and in eight of the 13 states with data for all three periods. Postpartum depression is associated with adverse maternal, infant, and child outcomes, including lower rates of breastfeeding initiation and shorter duration (*1*), poor maternal and infant bonding (*2*), and infant developmental disorders (*3*). The specific etiology of postpartum depression is unknown; however, risk factors include depression during pregnancy, low social support, stressful life events during pregnancy, preterm birth, and a traumatic birth experience (*4*). Contextual factors, such as the reduction in the birth rate of teens aged 15–19 years from 41.5 in 2007 to 24.2 per 1,000 females in 2014 and reduction in the preterm birth rate from 10.4% in 2007 to 9.5% in 2014 (*5*), reduction of women experiencing self-reported stressful life events in the year preceding birth by 0.54 percentage points per year from 2000 to 2010 (*6*), and an increase in antidepressant prescriptions to pregnant women from 0.7% in 2002–2006 to 2.1% in 2007–2010 (*7*) might have influenced the observed decline in PDS.

Postpartum depression is treatable with pharmacologic therapy and/or behavioral health interventions. However, depression is often underdiagnosed and untreated; nearly 60% of women with depressive symptoms do not receive a clinical diagnosis, and 50% of women with a diagnosis do not receive any treatment (*8*). Despite the observed decline, PDS remain common, affecting 11.5% of new mothers in 2012, with prevalence varying by reporting state and subgroups of women. These findings underscore the need for universal screening and appropriate treatment for pregnant and postpartum women, as recommended by the American College of Obstetricians and Gynecologists (ACOG) (*4*), the American Academy of Pediatrics (AAP) (*9*), and the U.S. Preventive Services Task Force.^§^ ACOG recommends that providers screen for depressive symptoms at least once during pregnancy or postpartum, using a validated screening tool (*4*). In addition, AAP recognizes that depression screening is part of family-centered well-child care, given pediatricians’ early access to the mother-infant duo (*9*). Collaboration between obstetric and pediatric providers is recommended for symptomatic women identified during newborn care (*4*,*9*). Recent efforts to address maternal depression include extending postpartum Medicaid coverage for women, integration of behavioral health services within primary care, and provider reimbursement for postpartum depression screening at well-baby visits.

The findings in this report are subject to at least three limitations. First, PDS are self-reported and might not represent a clinical diagnosis of depression. The PRAMS PDS two-item screener is based on the Patient Health Questionnaire-2. These questions with similar categorization schemes have a sensitivity of 58% and specificity of 85%, compared with clinical assessments of major depressive episodes (*10*); thus, the results in this report might underestimate the true prevalence of postpartum depression. Second, data might not be generalizable to states not included in this analysis or pregnancies that did not result in a live birth. Finally, PRAMS has limited data on mental health treatment, including antidepressant use; thus, mental health treatment over time could not be assessed in this report.

PRAMS data can be used to monitor progress toward meeting the *Healthy People 2020* objective to decrease the proportion of women delivering a live birth who experience PDS. Despite the observed decline in prevalence, approximately one in nine women experience PDS, with higher prevalence in certain states and subgroups of women. Ongoing surveillance and activities to promote appropriate screening, referral, and treatment are needed to reduce PDS among U.S. women. In addition, more research is needed to understand the etiology of postpartum depression.

**Summary**

**What is already known about this topic?**

Postpartum depressive symptoms (PDS) are common and are associated with adverse maternal and infant outcomes (e.g., lower breastfeeding initiation and duration and poor maternal and infant bonding). Postpartum depression is treatable.

**What is added by this report?**

This report provides recent state-specific trends in self-reported PDS. Among the 13 states with data for all three periods (2004, 2008, and 2012), self-reported prevalence of PDS declined from 14.8% in 2004 to 9.8% in 2012. During 2004–2012, statistically significant declines were observed in eight of 13 states (Alaska, Colorado, Georgia, Hawaii, Minnesota, Nebraska, Utah, and Washington), and no statistically significant changes in prevalence were observed in five states (Maine, Maryland, Oregon, Rhode Island, and Vermont). In 2012, the overall PDS prevalence was 11.5% for 27 states.

**What are the implications for public health practice?**

Despite the observed decline, PDS remain common. A developmental *Healthy People* *2020* objective is to decrease the proportion of women delivering a live birth who experience PDS. This report highlights the disparities in the prevalence of self-reported PDS by reporting state and subgroups of women. Ongoing surveillance and activities to promote universal screening followed by appropriate referral and treatment are needed to reduce PDS among U.S. women.

**TABLE 2:** Percentage of new mothers with postpartum depressive symptoms, by selected characteristics — Pregnancy Risk Assessment Monitoring System (PRAMS), 13 reporting states,* 2004, 2008, and 2012

Characteristic	2004 % (95% CI)	2008 % (95% CI)	2012 % (95% CI)	Linear trend^†^ p-value	Average annual percentage-point change during 2004–2012^§^
**Maternal age group (yrs)**					
≤19	24.6 (21.3–28.3)	21.4 (17.2–26.3)	18.3 (14.9–22.2)	0.016	-0.8
20–24	18.5 (16.7–20.5)	16.8 (14.6–19.2)	11.5 (9.8–13.4)	<0.001	−0.9
25–34	12.4 (11.4–13.6)	10.2 (9.1–11.3)	8.6 (7.7–9.7)	<0.001	-0.5
≥35	11.0 (9.3–13.0)	8.8 (7.5–10.4)	8.9 (7.2–10.8)	0.102	—^¶^
Maternal race/Ethnicity**					
White, Non-Hispanic	11.9 (10.9–12.9)	10.4 (9.4–11.4)	8.6 (7.6–9.6)	<0.001	-0.4
Black, Non-Hispanic	21.5 (19.0–24.2)	18.9 (14.8–23.9)	10.8 (8.5–13.7)	<0.001	-1.3
Hispanic	18.2 (15.9–20.9)	13.4 (11.5–15.6)	10.5 (8.7–12.5)	<0.001	-0.9
American Indian/Alaska Native	22.8 (18.7–27.5)	19.0 (16.2–22.1)	17.5 (14.1–21.6)	0.071	—^¶^
Asian/Pacific Islander	18.5 (16.1–21.2)	14.9 (12.5–17.6)	14.0 (11.7–16.7)	0.018	−0.5
Other	29.8 (19.6–42.6)	17.6 (11.4–26.3)	10.7 (7.5–15.0)	<0.001	−2.0
**Education level (yrs)**					
<12	23.6 (21.0–26.3)	20.2 (17.1–23.6)	13.4 (11.2–16.0)	<0.001	−1.2
12	17.4 (15.9–19.1)	14.9 (13.1–17.0)	12.3 (10.6–14.2)	<0.001	−0.6
>12	10.4 (9.5–11.4)	9.1 (8.2–10.2)	8.0 (7.2–8.9)	<0.001	−0.3
**Marital status**					
Unmarried	22.0 (20.3–23.9)	18.5 (16.4–20.7)	12.7 (11.3–14.2)	<0.001	−0.1
Married	11.5 (10.7–12.5)	9.4 (8.6–10.3)	8.4 (7.5–9.3)	<0.001	−0.2
**No. of previous live births**					
First birth	13.5 (12.3–14.8)	12.0 (10.6–13.6)	9.4 (8.3–10.6)	<0.001	−0.5
Second or later birth	15.7 (14.6–16.8)	13.0 (11.8–14.3)	10.0 (9.0–11.0)	<0.001	−0.7
**Postpartum smoking status**					
Nonsmoker	12.4 (11.6–13.3)	11.1 (10.1–12.1)	8.7 (8.0–9.5)	<0.001	−0.5
Smoker	26.3 (23.7–29.2)	21.8 (18.8–25.1)	17.7 (14.9–20.8)	<0.001	−1.1
**No. of stressful life events in 12 months before birth**					
None	7.3 (6.3–8.5)	5.6 (4.7–6.6)	6.4 (5.2–7.7)	0.268	—^¶^
1–2	12.2 (11.0–13.4)	11.4 (10.1–13.0)	8.0 (7.0–9.2)	<0.001	−0.5
3–5	24.0 (21.9–26.3)	20.2 (17.8–22.8)	14.4 (12.6–16.3)	<0.001	−1.2
6–13	37.3 (32.5–42.3)	34.0 (28.5–40.0)	24.2 (20.0–29.0)	<0.001	−1.6
**Gestational age and birthweight** ^††^					
Preterm	19.0 (16.6–21.6)	15.4 (13.4–17.7)	11.7 (9.8–13.8)	<0.001	−0.9
Term, low birthweight	20.4 (16.0–25.5)	19.1 (15.4–23.5)	17.6 (13.4–22.8)	0.412	—^¶^
Term, normal birthweight	14.2 (13.4–15.2)	12.0 (11.0–13.0)	9.5 (8.7–10.4)	<0.001	−0.6
**Infant admission to NICU at birth**					
No	14.0 (13.1–14.9)	11.7 (10.8–12.7)	9.5 (8.7–10.4)	<0.001	−1.0
Yes	20.5 (17.9–23.4)	18.6 (15.6–22.0)	12.5 (10.4–14.9)	<0.001	−0.5

**Abbreviations:** CI = confidence interval; NICU = neonatal intensive care unit.

* Included 13 states that had data for all three periods: Alaska, Colorado, Georgia, Hawaii, Maine, Maryland, Minnesota, Nebraska, Oregon, Rhode Island, Utah, Vermont, and Washington.

^†^ State-specific linear trends were assessed using logistic regression models among states with all 3 periods using year of birth as the predictor.

^§^ Unadjusted average annual percentage-point change in prevalence within selected characteristic during 2004–2012 was calculated using the beta coefficient of the infant's birth year from the linear model and the average percentage during 2004–2012.

^¶^ Average annual percentage-point change was not computed because linear trend was not significant.

** Vermont data do not include race/ethnicity, and were excluded from subgroup analysis.

^††^ Preterm birth: <37 weeks’ gestation; Term, low birthweight: ≥37 weeks’ gestation and <2,500 g; Term, normal birthweight: ≥37 weeks’ gestation and ≥2,500 g.

## Footnotes

* https://www.healthypeople.gov/2020/topics-objectives/topic/maternal-infant-and-child-health/objectives.

† https://www.cdc.gov/prams/methodology.htm.

§ https://www.uspreventiveservicestaskforce.org/Page/Document/UpdateSummaryFinal/depression-in-adults-screening.
